# What clinical practices for intensive care psychologists in France? A national survey

**DOI:** 10.1186/s13054-024-04987-z

**Published:** 2024-06-20

**Authors:** Alicia Landbeck, Arnaud Witt, Emilie Marty Petit, Emilie Aebischer, Anne-Laure Poujol, Stéphanie Nguyen, Etienne Simon, Pauline Bernigaud, Guillaume Thiery, Belaid Bouhemad, Alexandra Laurent

**Affiliations:** 1 Laboratoire Psy-DREPI, PSY-DREPI UR 7458, Université de Bourgogne Pôle AAFE, University of Burgundy, Esplanade Erasme, 21078 Dijon, France; 2 LEAD CNRS UMR 5022, University of Burgundy, Dijon, France; 3 Intensive Care Unit, Centre Hospitalier Intercommunal de Poissy and St Germain en Laye, Poissy, France; 4 Groupe Patient-Proche SRLF, Paris, France; 5 Intensive Care Unit, Centre Hospitalier Intercommunal de Créteil, Créteil, France; 6 École de Psychologues Praticiens, Institut Catholique de Paris, EA 7403, Paris, France; 7 Polyvalent Surgical Intensive Care Unit, Department of Anesthesia and Intensive Care, Hôpital de La Pitié-Salpêtrière, Assistance Publique-Hôpitaux de Paris, Paris, France; 8 Laboratory of Psychology, University of Franche-Comté, Besançon, France; 9 Intensive Care Unit, Saint Antoine Hospital, Assistance Publique -Hôpitaux de Paris, Paris, France; 10 Intensive Care Unit, CHU Saint Etienne, Saint-Étienne, France; 11 Department of Anesthesia and Intensive Care, CHU Dijon-Bourgogne, Dijon, France

The emotional stakes in intensive care units (ICUs), linked to the severity of the illness, make the presence of psychologists essential for patients, relatives, and healthcare professionals [1]. In France, ICU psychologists are recruited at the instigation of the head of the unit or facility, but the COVID-19 health crisis highlighted the importance of the psychological impact of intensive care [2] and led to the decree of April 26, 2022, on the operating conditions in ICUs, which requires the presence of psychologists in these units (https://www.legifrance.gouv.fr/eli/decret/2022/4/26/SSAH2206984D/jo/texte). However, little attention has been paid to their practice and role in the ICU [3]. The aim of our survey was to describe the practice of intensive care psychologists in France.

A questionnaire was designed by our team of researchers, psychologists, and ICU staff. It consisted of 72 questions (closed and open-ended questions),divided into five main themes: 1/ taking up a position in the ICU, 2/ working in the ICU with patients, relatives, and healthcare professionals, 3/ working conditions, 4/ professional resources, and 5/ initial training and training needs of the psychologist (Questionnaire in the supplementary material).

A total of 295 email addresses of ICU psychologists were identified within the 316 French healthcare institutions (IGAS—General Inspectorate of Social Affairs; 2021). Between February and April 2023, the psychologists identified were asked to complete the questionnaire via a LimeSurvey link. The responses were analyzed using descriptive analysis of quantitative and qualitative data.

Of the 295 psychologists listed, 153 (143 female and 10 male) responded to the questionnaire (Table 1). The psychologists worked in a unit with an average of 23 beds (standard deviation = 15). Although there appeared to be a relationship between the number of beds in the unit and the workload, the results showed a wide disparity between the number of beds and the amount of time psychologists spent in the ICU.

**Table 1:** Demographic characteristics and work context of ICU psychologists

Characteristics	Psychologists (n = 153)
*Age*	
21–29 years	26 (17%)
30–44 years	79 (52%)
45–60 years	40 (26%)
Over 60	8 (5%)
*Type*	
Female	143 (93%)
Male	10 (7%)
*Place of work*	
University hospital	78 (51%)
Private hospital/clinic	75 (49%)
*Experience*	
Less than one year	25 (16%)
Between one and five years	66 (43%)
Between six and 10 years	27 (18%)
Over 10 years	35 (23%)
*Contract type*	
Indefinite term	119 (78%)
Fixed-term	33 (21%)
Other	1 (1%)
*Type of ICU*	
Polyvalent	68 (44%)
Medical	42 (27%)
Surgical	36 (24%)
Pediatric	27 (18%)
Neonatal	20 (13%)
*Number of beds*	
Average (standard deviation)	23 (15)
*Working hours*	
Less than half-time	36 (23%)
Half-time	38 (25%)
More than half-time	33 (22%)
Full time	46 (30%)
*Average number (and range) of beds in relation to the number of hours worked *(r = 0.14, *p* = 0.086)	
Less than half-time	20 (range: 6–63)
Half-time	21 (range: 2–62)
More than half-time	25 (range: 6–60)
Full time	26 (range: 6–84)
*Primary position*	
ICU exclusively	76 (50%)
Several departments, including ICU	66 (43%)
Occasional interventions	11 (7%)
*Average number of beds in relation to the number of hours worked *(r = 0.27, *p* = 0.017)* for psychologists who work in ICU exclusively *(n = 76)	
Less than half-time (n = 14; 18%)	24 (range: 11–60)
Half-time (n = 29; 38%)	24 (range: 8–62)
More than half-time (n = 11; 14%)	35 (range: 20–60)
Full time (n = 22; 29%)	32 (range: 16–84)
*Workspace*	
Office	71 (63%)
No office	40 (26%)
Shared office	42 (37%)
*Regular clinical practice (more than once a week) with*	
Patients	137 (90%)
Families	128 (84%)
Healthcare professionals	102 (67%)
Institutional meetings (staff)	111 (73%)
*Wish to remain in intensive care*	
Yes	130 (85%)
No	23 (15%)
*Theoretical approaches*	
Psychoanalytical	123 (80%)
Systemic	47 (31%)
Cognitive-behavioral	31 (20%)
Developmental	22 (14%)
Cognitive and neuropsychology	18 (12%)
Social psychology	11 (7%)
Work and organizational psychology	10 (6%)
At least two of the above approaches	69 (45%)

ICU psychologists interviewed worked the majority of psychologists surveyed interacted with patients (90%), families (84%), and healthcare professionals (67%) more than once a week. Their psychological practice is based on the theoretical models of psychoanalysis (80%). Only 63% of the psychologists had an office or a dedicated workspace. In addition, the head of the ICU defined the psychologist’s tasks in 64% of the cases. However, these tasks remained unclear for 47.5% of these psychologists. Almost half of the psychologists (44.4%) wanted to continue working in the ICU, but 13.7% of them were considering leaving their position and 15% said they were planning to leave the hospital.

Our survey shows that ICU psychologists developed a wide range of specific tools and devices (Additional file 1: Tables S2, S3, S4). In this context of acute care and serious illness, the psychologist must meet and engage with the patient, family or healthcare professionals to understand their difficulties and adjust to their needs. These various discussions often took place in the common areas of the ICU (patients’ rooms, corridors, rest rooms, etc.). These “informal” encounters, without an appointment, represented a “therapeutic priming” which required flexible modes of interviewing adapted to the diversity of places and people involved.

With patients, psychologists developed a clinical approach based on a variety of interpersonal relationships using slates, eye-tracking, touch, gaze, and psycho-corporal practices (relaxation, hypnosis, virtual reality, etc.). With families, they relied on pamphlets to explain the care environment to the family [4] and especially to visiting children [5]. They also used diaries to support the family and the patient in the ICU or during post-ICU consultations.

The involvement of psychologists with patients and their families extended well beyond the ICU. Indeed, the psychological impact of the ICU after discharge led them to develop their practice in other hospital units by offering post-ICU counseling, thus linking the different phases of hospitalization and the post-ICU period.

Finally, an explicit request for the psychologist by the healthcare professionals was rare. As a result, the presence of psychologists dedicated to the ICU seems crucial as it allows psychologists to fully understand the working environment of healthcare professionals and to develop relationships with the team over time, thus creating a climate of trust in which to make a request [2].

The main limitations of this study are, on the one hand, its observational design, which does not allow us to conclude whether one psychological practice is more relevant than another. Second, the sample remains selective, as not all psychologists working in an ICU participated in the survey. However, our results highlight the richness and diversity of psychological interventions in the ICU and post-ICU and underline the importance of developing studies on psychological devices/tools in order to better support and recognize the role of the ICU psychologist.

### Supplementary Information

**Additional file 1: Table S1.** Psychologists’ interventions with patients expressed as a percentage (calculated on the basis of the responses “regularly” and “often” based on the total number of participants interacting with patients.** Table S2.** Psychologists’ interventions with families expressed as a percentage (calculated on the basis of the responses “regularly” and “often” based on the total number of participants interacting with patients).** Table S3.** Psychologists’ interventions with healthcare professionals expressed as a percentage (calculated on the basis of the responses “regularly” and “often” based on the total number of participants interacting with patients)

## Data Availability

The datasets used and/or analyzed during the current study are available from the corresponding author on reasonable request.
