# Supplementary material for: What clinical practices for intensive care psychologists in France? A national survey
Source: Crit Care. 2024 Jun 20;28:204. doi: 10.1186/s13054-024-04987-z (PMC11188499; doi:10.1186/s13054-024-04987-z)
Supplement: Supplementary file 1 — Additional file 1: Table S1. Psychologists’ interventions with patients expressed as a percentage (calculated on the basis of the responses “regularly” and “often” based on the total number of participants interacting with patients. Table S2. Psychologists’ interventions with families expressed as a percentage (calculated on the basis of the responses “regularly” and “often” based on the total number of participants interacting with patients). Table S3. Psychologists’ interventions with healthcare professionals expressed as a percentage (calculated on the basis of the responses “regularly” and “often” based on the total number of participants interacting with patients) [file 13054_2024_4987_MOESM1_ESM.docx]

**Electronic supplementary material**

[Questionnaire 2](#_Toc169369559)

[Table S1: Psychologists’ interventions with patients expressed as a percentage (calculated on the basis of the responses “regularly” and “often” based on the total number of participants interacting with patients 16](#_Toc169369560)

[Table S2 : Psychologists’ interventions with families expressed as a percentage (calculated on the basis of the responses “regularly” and “often” based on the total number of participants interacting with patients) 17](#_Toc169369561)

[Table S3 : Psychologists’ interventions with healthcare professionals expressed as a percentage (calculated on the basis of the responses “regularly” and “often” based on the total number of participants interacting with patients) 18](#_Toc169369562)

## **Questionnaire**

**Section A: SOCIO-DEMOGRAPHIC INFORMATION**

**A1. Your age group:**

21-29 years ☐

30-44 years ☐

45-60 years ☐

Over 60 ☐

**A2. Gender:**

Female ☐

Male ☐

Do not wish to answer ☐

**Section B: YOUR POSITION IN THE ICU**

**B1. How long have you worked in the ICU?**

Less than one year ☐

Between 1 and 5 years ☐

Between 6 and 10 years ☐

Over 10 years ☐

**B2. In which structure do you work?**

University hospital ☐

Hospital ☐

Private clinic☐

Other ☐

**B3. In which region do you work?**

Auvergne-Rhône-Alpes ☐

Bourgogne-Franche-Comté ☐

Bretagne ☐

Centre-Val de Loire ☐

Grand Est ☐

Haut de France ☐

Ile-de-France ☐

Normandie ☐

Nouvelle-Aquitaine ☐

Occitanie ☐

Pays de la Loire ☐

 Provence-Alpes-Côte d'Azur ☐

**B4. In which ICU department(s) do you work?**

Medical ICU ☐

Multi-purpose ICU ☐

Surgical ICU ☐

Pediatric ICU ☐

Neonatal ICU ☐

**B5. If specialized department, please specify:**

**B6. What employment contract do you have?**

Permanent contract ☐

Fixed-term contract ☐

Other ☐

**B7. What is your hourly rate?**

Full time ☐

More than half-time ☐

Half-time ☐

Less than half-time ☐

**B8. Is your position as a psychologist specifically dedicated to the ICU?**

My position is dedicated exclusively to the ICU ☐

My position is dedicated to several departments, including the ICU  ☐

I occasionally intervene in the ICU ☐

**B9. On an average of one month, how often do you intervene?**

Less than once a month ☐

1 to 2 times a month ☐

3 to 6 times a month ☐

7 to 10 times a month ☐

**B10. Who asks you to intervene in the ICU?**

More than 10 times ☐

Nurses ☐

Nursing assistants ☐

Doctors ☐

Executives ☐

**B11. Number of beds in the ICU(s):**

 *Indicate the total number of beds under your care:

**B12. Has there been a psychologist in the department before you?**

Yes ☐

No ☐

I don't know ☐

**B13. How long was he or she in the department?**

Less than one year ☐

Between 1 and 5 years ☐

More than 5 years ☐

I don't know ☐

**B14. Why did you take up this position?**

|  | Not at all | Rather no | Rather Yes | Yes, absolutely |
| --- | --- | --- | --- | --- |
| Clinical, hospital, and ICU interests | ☐ | ☐ | ☐ | ☐ |
| Job opportunity, job market, contractual benefit(s) | ☐ | ☐ | ☐ | ☐ |

**Section C: CLINICAL PRACTICE IN THE ICU**

**C1. On average, how often do you work with patients, relatives, and medical staff?**

|  | Never | About once a month or less | About once a week or every 2 weeks | More than once a week |
| --- | --- | --- | --- | --- |
| With patients | ☐ | ☐ | ☐ | ☐ |
| With relatives | ☐ | ☐ | ☐ | ☐ |
| With medical staff | ☐ | ☐ | ☐ | ☐ |

**C2. In what context do you work with patients?**

|  | Never | Rarely | Regularly | Often | Not concerned |
| --- | --- | --- | --- | --- | --- |
| In the room when patients are able to communicate (verbally or non-verbally) | ☐ | ☐ | ☐ | ☐ | ☐ |
| In the room when patients are unable to communicate | ☐ | ☐ | ☐ | ☐ | ☐ |
| During care and/or nursing | ☐ | ☐ | ☐ | ☐ | ☐ |
| At the end of life | ☐ | ☐ | ☐ | ☐ | ☐ |
| Post-ICU consultations (multidisciplinary consultations for patients/relatives post-ICU) | ☐ | ☐ | ☐ | ☐ | ☐ |
| Post-ICU following change of medical service or return home | ☐ | ☐ | ☐ | ☐ | ☐ |
| Other | ☐ | ☐ | ☐ | ☐ | ☐ |

**C3. You answered “Other” in the previous section. Could you please specify?**

**C4. Do you use specific tools (logbooks, scales, tests, booklets, etc.)?**

Yes ☐

No ☐

**C5. If yes, Please specify:**

**C6. What kind of difficulties(s) have you encountered with patients?**

|  | Never | Rarely | Regularly | Often |
| --- | --- | --- | --- | --- |
| Absence of requests | ☐ | ☐ | ☐ | ☐ |
| Patients' lack of understanding of the psychologist's role | ☐ | ☐ | ☐ | ☐ |
| Team’s misunderstanding of psychologist's role | ☐ | ☐ | ☐ | ☐ |
| Refusal to see the psychologist | ☐ | ☐ | ☐ | ☐ |
| Unsuitable room for your practice | ☐ | ☐ | ☐ | ☐ |
| Patient transferred | ☐ | ☐ | ☐ | ☐ |
| Interruption of your practice by the care team | ☐ | ☐ | ☐ | ☐ |
| Communication difficulties/impossibility | ☐ | ☐ | ☐ | ☐ |
| Other | ☐ | ☐ | ☐ | ☐ |

**C7. You answered “Other” in the previous section. Could you please specify?**

**C8. In general, your interactions with patients are aimed at:**

Evaluative ☐

Therapeutic ☐

Support ☐

Other ☐

If other, please specify:

**C9. In what context do you work with relatives?**

|  | Never | Rarely | Regularly | Often | Not concerned |
| --- | --- | --- | --- | --- | --- |
| During scheduled interviews in a dedicated area | ☐ | ☐ | ☐ | ☐ | ☐ |
| During scheduled in-room interviews | ☐ | ☐ | ☐ | ☐ | ☐ |
| During spontaneous interviews in rooms or dedicated areas | ☐ | ☐ | ☐ | ☐ | ☐ |
| During departmental discussions (corridor, waiting  room...) | ☐ | ☐ | ☐ | ☐ | ☐ |
| During family interviews with the medical team  (announcement, interview, etc.) | ☐ | ☐ | ☐ | ☐ | ☐ |
| During telephone interviews | ☐ | ☐ | ☐ | ☐ | ☐ |
| Post-ICU consultations (multidisciplinary consultations for patients/relatives post-ICU) | ☐ | ☐ | ☐ | ☐ | ☐ |
| Post-ICU after transfer or return home | ☐ | ☐ | ☐ | ☐ | ☐ |
| As part of discussion groups | ☐ | ☐ | ☐ | ☐ | ☐ |
| As part of a workshop(s) | ☐ | ☐ | ☐ | ☐ | ☐ |
| During interactions with child visitors | ☐ | ☐ | ☐ | ☐ | ☐ |
| Other | ☐ | ☐ | ☐ | ☐ | ☐ |

**C10. Can you specify the type(s) of workshop(s) or group(s) you use?**

**C11. You answered “Other” in the previous section. Could you please specify?**

**C12. Do you use specific tools (logbooks, scales, tests, booklets, etc.)?**

Yes ☐

No ☐

**C13. If yes, please specify:**

**C14.  What kind of difficultie(s) have you encountered with relatives?**

|  | Never | Rarely | Regularly | Often |
| --- | --- | --- | --- | --- |
| Absence of requests | ☐ | ☐ | ☐ | ☐ |
| Family misunderstanding of the psychologist's role | ☐ | ☐ | ☐ | ☐ |
| Team misunderstanding of psychologist's role | ☐ | ☐ | ☐ | ☐ |
| Inappropriate requests | ☐ | ☐ | ☐ | ☐ |
| Inappropriate facilities | ☐ | ☐ | ☐ | ☐ |
| Missed appointments | ☐ | ☐ | ☐ | ☐ |
| Refusal to see the psychologist | ☐ | ☐ | ☐ | ☐ |
| Other | ☐ | ☐ | ☐ | ☐ |

**C15. You answered “Other” in the previous section. Could you please specify?**

**C16. In general, your interaction with relatives are aimed at:**

Informative ☐

Preventive ☐

Evaluative ☐

Therapeutic ☐

Supportive ☐

Other ☐

If other, please specify:

**C17. When you work with medical personnel, you perform:**

|  | Never | Rarely | Regularly | Often | Not Concerned |
| --- | --- | --- | --- | --- | --- |
| Informal exchanges within the service | ☐ | ☐ | ☐ | ☐ | ☐ |
| Punctual one-to-one meetings | ☐ | ☐ | ☐ | ☐ | ☐ |
| Individual psychological support | ☐ | ☐ | ☐ | ☐ | ☐ |
| Training courses | ☐ | ☐ | ☐ | ☐ | ☐ |
| Discussion | ☐ | ☐ | ☐ | ☐ | ☐ |
| Sessions | ☐ | ☐ | ☐ | ☐ | ☐ |
| Analysis of practice | ☐ | ☐ | ☐ | ☐ | ☐ |
| Debriefing following a difficult situation within the service | ☐ | ☐ | ☐ | ☐ | ☐ |
| Workshops | ☐ | ☐ | ☐ | ☐ | ☐ |
| Other | ☐ | ☐ | ☐ | ☐ | ☐ |

**C18. Can you specify the type(s) of workshop(s) or group(s) you use?**

**C19. You answered "Other" in the previous section. Could you please specify?**

**C20. Do you use specific tools (logbooks, scales, tests, booklets...)?**

Yes  ☐

 No ☐

**If yes, please, specify:**

**C24. In general, your interactions with medical personnel are intended to be:**

Informative   ☐

Preventive  ☐

Evaluative  ☐

Therapeutic  ☐

Supportive  ☐

Other (please, specify)  ☐

**C25. Do you participate in the following multidisciplinary activities?**

|  | Never | Rarely | Regularly | Often | Not concerned |
| --- | --- | --- | --- | --- | --- |
| Staff meetings | ☐ | ☐ | ☐ | ☐ | ☐ |
| Shift changes | ☐ | ☐ | ☐ | ☐ | ☐ |
| In-room multidisciplinary interviews | ☐ | ☐ | ☐ | ☐ | ☐ |
| RMM/CREX | ☐ | ☐ | ☐ | ☐ | ☐ |
| Organ harvesting consultations | ☐ | ☐ | ☐ | ☐ | ☐ |
| Announcement talks | ☐ | ☐ | ☐ | ☐ | ☐ |
| Meetings to limit or discontinue care | ☐ | ☐ | ☐ | ☐ | ☐ |
| Other | ☐ | ☐ | ☐ | ☐ | ☐ |

**C26. You answered "Other" in the previous section. Could you please specify?**

**Section D: YOUR WORKING CONDITIONS**

**D1. To what extent do you agree with the following statement?**

|  | Not at all | Rather no | Rather Yes | Yes, absolutely |
| --- | --- | --- | --- | --- |
| Overall, I’m satisfied with my work | ☐ | ☐ | ☐ | ☐ |

**D2. Currently, you can say that you feel:**

|  | Strongly disagree | Somewhat disagree | Somewhat agree | Strongly agree | Do not wish to answer |
| --- | --- | --- | --- | --- | --- |
| Supported/integrated by the head of the ICU | ☐ | ☐ | ☐ | ☐ | ☐ |
| Assisted/guided by the head of the ICU | ☐ | ☐ | ☐ | ☐ | ☐ |
| Recognized in your position by the head of the ICU | ☐ | ☐ | ☐ | ☐ | ☐ |

**D3. Currently, you can say that you feel:**

|  | Strongly disagree | Somewhat disagree | Somewhat agree | Strongly agree | Do not wish to answer |
| --- | --- | --- | --- | --- | --- |
| Supported/integrated by managers | ☐ | ☐ | ☐ | ☐ | ☐ |
| Helped/guided by managers | ☐ | ☐ | ☐ | ☐ | ☐ |
| Recognized in your position by managers | ☐ | ☐ | ☐ | ☐ | ☐ |

**D4. Currently, you can say that you feel:**

|  | Strongly disagree | Somewhat disagree | Somewhat agree | Strongly agree | Do not wish to answer |
| --- | --- | --- | --- | --- | --- |
| Supported/integrated by nurses and nursing assistants | ☐ | ☐ | ☐ | ☐ | ☐ |
| Helped/guided by nurses and nursing assistants | ☐ | ☐ | ☐ | ☐ | ☐ |
| Recognized in your position by nurses and nursing assistants | ☐ | ☐ | ☐ | ☐ | ☐ |

**D5. Currently, you can say that you feel:**

|  | Strongly disagree | Somewhat disagree | Somewhat agree | Strongly agree | Do not wish to answer |
| --- | --- | --- | --- | --- | --- |
| Supported/integrated by doctors | ☐ | ☐ | ☐ | ☐ | ☐ |
| Helped/guided by doctors | ☐ | ☐ | ☐ | ☐ | ☐ |
| Recognized in your position by doctors | ☐ | ☐ | ☐ | ☐ | ☐ |

**D6. Do you have a dedicated office or workspace?**

Yes ☐

No ☐

**D7. What kind?**

Private ☐

Shared ☐

**D8. Does this situation suit you?**

Yes ☐

No ☐

**D9. Your work objectives were given to you by...**

|  | Yes | No |
| --- | --- | --- |
| The head of ICU | ☐ | ☐ |
| Executive(s) | ☐ | ☐ |
| Administrative staff | ☐ | ☐ |
| Psychologist colleagues | ☐ | ☐ |
| College or psychologist’s department | ☐ | ☐ |
| Other hospital staff | ☐ | ☐ |
|  |  |  |

**D10. Your work objectives have been clearly communicated to you by:**

|  | Not Concerned | Strongly disagree | Somewhat disagree | Somewhat agree | Strongly agree |
| --- | --- | --- | --- | --- | --- |
| The head of ICU | ☐ | ☐ | ☐ | ☐ | ☐ |
| Manager(s) | ☐ | ☐ | ☐ | ☐ | ☐ |
| Administrative staff | ☐ | ☐ | ☐ | ☐ | ☐ |
| Psychology colleagues | ☐ | ☐ | ☐ | ☐ | ☐ |
| By the college or psychologists' department | ☐ | ☐ | ☐ | ☐ | ☐ |
| Other hospital staff | ☐ | ☐ | ☐ | ☐ | ☐ |

**D11. Are your work objectives reviewed regularly? (At the end of a fixed-term contract or annually for permanent staff)**

Yes ☐

No ☐

**D12. Who conducts the assessment/ evaluation?**

Head of ICU Executives ☐

College of psychologists Human resources manager ☐

Plant Manager ☐

**D13. How often have you seriously considered leaving your current job**

**in intensive care?**

Never ☐

Rarely ☐

Sometimes ☐

Quite often ☐

Very often ☐

**D14. Are you planning to leave the ICU?**

Yes ☐

 No ☐

**Section E: RESOURCES**

**E1. What resources help you in your ICU practice?**

Reading books ☐

Reading research articles ☐

Academic training ☐

Supervision/Intervision ☐

Individual care ☐

Participation in conferences and meetings ☐

Intensive care congresses (SRLF, SFAR, ESICM...) ☐

Psychologist associations ☐

Hospital colleagues ☐

Out-of-hospital colleagues ☐

Hierarchy (manager, department head, etc.) ☐

Medical professional ☐

Paramedical professional ☐

Administrative staff ☐

**E2. Regarding supervision/intervision:**

This is your own initiative ☐

You benefit from this approach thanks to your department or institution ☐

Other ( please specify) ☐

**E3. Are you a member of a resuscitation think tank?**

Yes ☐

No ☐

**E4. Which one?**

**Section F: TRAINING AND TRAINING NEEDS**

**F1. In what year did you become a licenced psychologist? (YYYY)**

**F2. Approach(es) on which you rely as part of your resuscitation practice:**

Developmental ☐

Psychodynamic ☐

Cognitive-behavioral ☐

Cognitive and neuropsychology ☐

Social psychology ☐

Work and organizational psychology ☐

Systemic ☐

Other ☐

If Other, please specify:

**F3. Do you need additional knowledge/skills to work in intensive care?**

Not at all   ☐

Rather no  ☐

Rather yes  ☐

Yes, absolutely ☐

**F4. For what critical care need(s)?**

|  | Not at all | Rather no | Rather Yes | Yes, absolutely |
| --- | --- | --- | --- | --- |
| Care team support | ☐ | ☐ | ☐ | ☐ |
| Patient support | ☐ | ☐ | ☐ | ☐ |
| Supervision/ practice analysis | ☐ | ☐ | ☐ | ☐ |

**F5. What type of training do you have in mind?**

|  | Not at all | Rather no | Rather Yes | Yes, absolutely |
| --- | --- | --- | --- | --- |
| University degree(s) | ☐ | ☐ | ☐ | ☐ |
| Conferences, scientific days | ☐ | ☐ | ☐ | ☐ |
| Integration into workgroups | ☐ | ☐ | ☐ | ☐ |
| Supervision/ practice analysis | ☐ | ☐ | ☐ | ☐ |
| Other : | ☐ | ☐ | ☐ | ☐ |

**F6. You answered "Other" in the previous section. Could you please specify?**

**F7. On what topic(s) would you like to receive training?**

**The somatic-psychic link / body awareness**

Trauma clinic ☐

Bereavement ☐

Delusions, psychiatric disorders ☐

Coma and coma awakening ☐

Anxiety disorders ☐

Organ harvesting process ☐

Medical vocabulary and terminology related to rescuscitation ☐

Burnout ☐

Quality of life and working conditions (QLWC) ☐

Teamwork and conflict management ☐

Other  ☐

**F8. Have you worked in departments/structures other than the ICU?**

Yes ☐

 No ☐

**F9. In which sector(s)/service(s)?**

 Hospital/Clinic in a somatic care unit ☐

Other  ☐

**F10. Do you participate in research activities (publications, communications, esta**

**blishment of research within the department)?**

Yes ☐

No ☐

**F11. About these research activities :**

Research conducted by the doctors or nurses in your department ☐

Research conducted by a humanities researcher with whom you are collaborating ☐

Research for your thesis ☐

Research on your own initiative or as part of a training course (university diploma, etc.) ☐

Other (please, specify) : ☐

**Section G: TO FINISH**

**G1. Do you have any clarifications, observations, or reflections that you would like to share and that were not included in the questionnaire?**

## **Table S1: Psychologists’ interventions with patients expressed as a percentage (calculated on the basis of the responses “regularly” and “often” based on the total number of participants interacting with patients**

| ***Patient interventions*** | n=149 |
| --- | --- |
| In room when patient is able to communicate (verbally or otherwise) | 97.8% |
| End-of-life care | 80% |
| In room when patient is unable to communicate | 63.9% |
| Post-ICU: other department or at home | 63.2% |
| Post-ICU facilities (multidisciplinary consultations for patient/relative) | 35.4% |
| ***Aims of patient intervention*** |  |
| Assistance and support in case of distress or psychological disorders | 96,6% |
| Therapeutic (psychological follow-up) | 65,8% |
| Assessment of psychological disorders | 64,4% |
| Preventive (interview to prevent the risk of disorders such as anxiety, depression, PTSD) | 56,4% |
| Informative, to understand the psychological impact of intensive care | 39% |
| ***Difficulties encountered with patients*** |  |
| Interruption of psychological interventions | 51,7% |
| Communication difficulties or inability to communicate | 51,7% |
| Transferred patient | 45% |
| No request | 38,2% |
| Patients’ lack of understanding of the psychologist’s role | 19,5% |
| Room unsuitable for interventions | 19,5% |
| The team’s lack of understanding of the psychologist’s role | 15,4% |
| Refusal to see the psychologist | 14,9% |

## **Table S2 : Psychologists’ interventions with families expressed as a percentage (calculated on the basis of the responses “regularly” and “often” based on the total number of participants interacting with patients)**

| ***Families interventions*** | ***n=149*** |
| --- | --- |
| Interviews in a dedicated area | 75% |
| Visiting children | 74% |
| Interviews or discussions within the unit | 69.8% |
| Participation in family meetings with the medical team | 50% |
| Telephone interviews | 43.6% |
| In-room interviews | 31,8% |
| Post-ICU facilities (on-demand interviews or multidisciplinary counseling for patients/relatives) | 29.6% |
| Group sessions | 6.7% |
| ***Aims of family intervention*** |  |
| Assistance and support in case of distress or psychological disorders | 98% |
| Therapeutic (psychological follow-up) | 51% |
| Assessment of psychological disorders | 41% |
| Preventive (interview to prevent the risk of disorders e.g. anxiety, depression, PTSD, complicated grief) | 59% |
| Informative, to understand the psychological impact of intensive care | 55% |
| ***Difficulties encountered with families*** |  |
| No requests | 36,2% |
| Other (please specify) | 11,4% |
| Refusal to see the psychologist | 10,8% |
| Lack of understanding of the psychologist’s place and role | 10,8% |
| No-shows | 6,7% |

## **Table S3 : Psychologists’ interventions with healthcare professionals expressed as a percentage (calculated on the basis of the responses “regularly” and “often” based on the total number of participants interacting with patients)**

| ***Healthcare professionals interventions*** | **n=139** |
| --- | --- |
| Interviews (formal and informal) | 97% |
| Debriefings | 51.1% |
| Training | 35% |
| Discussion groups | 18.5% |
| Workshops | 6.3% |
| ***Aims of healthcare professionals intervention*** |  |
| Assistance and support in case of distress or psychological disorders | 84,2% |
| Preventive (interview to prevent the risk of disorders e.g. burnout, anxiety, depression, PTSD) | 60,4% |
| Informative to understand the psychological impact of intensive care | 77% |
| Therapeutic (psychological follow-up) | 11,5% |
| Assessment of psychological disorders | 15,1% |
| ***Difficulties encountered with healthcare professionals*** |  |
| No requests | 39,1% |
| Difficulties in setting up new systems | 32,6% |
| Lack of understanding of the psychologist’s place and role | 16,7% |
| Refusal to participate | 15,1% |
| No-shows | 5,8% |
